# Harmonizing Scale for Intelligent Sensors: Resolution-Conditioned Adaptation of Vision Foundation Models for Crowd Counting

**DOI:** 10.3390/s26103047

**Published:** 2026-05-12

**Authors:** Huan Xu, Zhiheng Chen, Sirou Shen, Haibin You, Chih-Cheng Chen

**Affiliations:** 1 School of Computer Engineering, Jimei University, Xiamen 361021, China; xuhuan@jmu.edu.cn (H.X.); 202221335027@jmu.edu.cn (Z.C.); 202421335009@jmu.edu.cn (S.S.); evecat@jmu.edu.cn (H.Y.); 2 Department of Automatic Control Engineering, Feng Chia University, Taichung 40724, Taiwan

**Keywords:** crowd counting, scale variation, selective inheritance learning, foundation models

## Abstract

Scale variation remains a fundamental challenge for intelligent surveillance sensors in crowd-counting and localization tasks. While recent selective inheritance methods have shown promise through multi-resolution feature fusion, they typically rely on conventional CNN backbones with limited representation capacity. Foundation models such as DINOv3 offer powerful self-supervised representations, yet directly applying frozen DINOv3 features to selective scale-aware counting is non-trivial: these features are semantically strong but not directly aligned with density estimation or scale-conditioned inheritance requirements. In this paper, we propose D3-CalibCount, a trainable-parameter-efficient framework for adapting frozen DINOv3 representations to selective scale-aware crowd counting and localization. We introduce a lightweight Scale Harmonization Adapter (SHA) that performs resolution-conditioned feature calibration, transforming generic DINOv3 representations into scale-selective counting features suitable for progressive inheritance across resolution levels. Extensive experiments on three widely used benchmarks show consistent improvements over selective inheritance baselines, especially under severe scale variation. From a deployment perspective, the method reduces the trainable portion of the network, while the frozen DINOv3-L backbone still introduces a higher inference cost than lighter CNN baselines. The target application scenario is camera-based intelligent surveillance sensing, where crowd density estimation is performed from visual sensor inputs at GPU-backed monitoring nodes. These results suggest that lightweight adaptation of frozen foundation features is a practical direction for crowd counting and other dense prediction tasks.

## 1. Introduction

Crowd counting and localization have emerged as critical computer vision tasks with wide-ranging applications in public safety monitoring, urban planning, traffic management, and event organization. The ability to accurately estimate crowd density and locate individual instances enables intelligent systems to detect anomalies, optimize resource allocation, and ensure public safety in densely populated environments. Recent advances in deep learning have significantly improved counting accuracy; yet, the fundamental challenge of scale variation—where objects of vastly different sizes coexist within a single scene—remains a persistent bottleneck that limits practical deployment.

Scale variation in crowd counting manifests in multiple forms: perspective distortion causes objects at different depths to appear at drastically different scales, varying camera viewpoints introduce geometric distortions, and the inherent diversity of crowd distributions leads to clusters of different densities. This multi-faceted challenge is particularly acute in real-world scenarios where a single image may contain both distant individuals occupying only a few pixels and nearby subjects spanning hundreds of pixels. Traditional single-scale approaches struggle to capture this diversity, often excelling at one scale range while failing at others, resulting in systematic undercounting or overcounting depending on the predominant object size.

To address scale variations, researchers have explored various multi-scale fusion strategies. Early methods, such as MCNN [1], employed multi-column architectures with different receptive fields, while more recent approaches, such as FPN-based designs [2], aggregate features across resolution hierarchies. However, these methods typically apply uniform optimization objectives across all scales, forcing each resolution to handle the entire scale spectrum, which leads to mutual suppression and degraded feature discriminability. Recent selective inheritance methods, exemplified by STEERER [3], have made progress by introducing scale-customized feature selection and progressive inheritance from lower to higher resolutions. These approaches typically rely on conventional CNN backbones (e.g., HRNet, VGG) that are either trained from scratch or initialized with ImageNet pre-training. While effective, such backbones have limited representation capacity and may struggle to capture the rich semantic and structural information needed for robust scale-aware counting across diverse scenarios.

The recent emergence of vision foundation models, particularly self-supervised learning approaches such as DINOv3 [4], presents new opportunities for addressing scale variation. DINOv3 is trained on massive, diverse datasets through self-distillation, learning rich visual representations that capture semantic, geometric, and structural information without task-specific supervision. However, directly applying frozen DINOv3 features to selective scale-aware counting is non-trivial. While these representations are semantically strong and exhibit remarkable generalization across domains, they are not directly optimized for crowd density estimation or selective scale-aware inheritance. Specifically, frozen DINOv3 features exhibit a task mismatch: (1) they prioritize global semantic understanding over local density-sensitive responses required for counting; (2) their token representations do not naturally align with crowd localization requirements; and (3) the feature statistics differ from those of conventional counting backbones, requiring careful adaptation for scale-conditioned inheritance. This gap between foundation features and counting-specific needs motivates the design of specialized adaptation mechanisms.

This work addresses a previously underexplored question: how to transform frozen foundation features into scale-selective counting representations without sacrificing parameter efficiency or generalization? We propose D3-CalibCount (**D**INOv**3**-based **Calib**rated **Count**ing), a parameter-efficient framework that adapts frozen DINOv3 representations for selective scale-aware crowd counting and localization. Our primary contribution is to show how a frozen vision foundation model can be introduced into the selective inheritance paradigm through resolution-conditioned adaptation, rather than to redefine selective inheritance itself. Concretely, we inherit from STEERER the progressive multi-resolution learning framework, the PWSP/MSIL supervision strategy, and the coarse-to-fine inheritance order, while the frozen DINOv3 backbone integration, the SHA module, and the corresponding feature-calibration pipeline are newly introduced in this work. Our key insight is that while selective inheritance provides an effective mechanism for scale-aware feature aggregation, its success critically depends on whether the underlying features are suitable for current-scale density estimation and cross-scale inheritance. Frozen DINOv3 features, despite their semantic richness, require resolution-conditioned calibration to align with these dual requirements. Rather than fine-tuning the entire foundation model, we introduce lightweight adaptation modules that preserve DINOv3’s generalization capability while enabling task-specific feature transformation.

To address this challenge, we introduce the Scale Harmonization Adapter (SHA), a lightweight module that performs resolution-conditioned calibration of frozen DINOv3 features. Unlike generic adapter designs, SHA is specifically tailored for selective scale-aware counting: it transforms foundation features into representations that are simultaneously suitable for current-resolution density estimation and forward-compatible for inheritance to higher resolutions. The adapter employs a dual-path architecture: a Current-Resolution Harmonization Path that extracts density-sensitive responses appropriate for the current scale range, and a Forward-Compatible Inheritance Path that preserves complementary features valuable for finer-scale analysis. A learnable soft-mask generator dynamically balances these paths based on local feature characteristics, enabling adaptive feature selection that aligns with the selective inheritance principle. This design ensures that frozen DINOv3 features are effectively adapted without requiring full model fine-tuning, maintaining parameter efficiency and cross-dataset generalization.

Our approach offers several advantages over existing methods. First, by leveraging frozen DINOv3 representations, we preserve the model’s generalization capability while enabling efficient task-specific adaptation. Second, the Scale Harmonization Adapter provides a trainable-parameter-efficient solution, requiring only 28.4 M trainable parameters compared with 65.8 M for methods that fine-tune entire backbones. Third, the resolution-conditioned calibration design ensures that foundation features are effectively transformed for selective scale-aware counting. Extensive experiments on three widely used benchmarks (ShanghaiTech, UCF-QNRF, and JHU-Crowd++) demonstrate consistent improvements over selective inheritance baselines, with notable gains on challenging scenarios featuring severe scale variations. These results validate the effectiveness of foundation-feature adaptation for scale-variant crowd counting and localization.

The main contributions of this work are summarized as follows:

- We introduce frozen foundation representations into the selective inheritance paradigm for crowd counting and show that resolution-conditioned calibration is needed to make them compatible with density estimation and cross-scale inheritance.
- We introduce the Scale Harmonization Adapter (SHA), a lightweight module that performs resolution-conditioned feature calibration, which transforms generic DINOv3 representations into scale-selective counting features suitable for progressive inheritance.
- We demonstrate that trainable-parameter-efficient adaptation of frozen foundation features can consistently improve crowd counting and localization under severe scale variation, while also exposing an explicit accuracy-cost trade-off at deployment time.
- Extensive experiments on three widely used benchmarks (ShanghaiTech, UCF-QNRF, and JHU-Crowd++) demonstrate consistent improvements over selective inheritance baselines, with notable gains on challenging scenarios involving severe scale variations.

## 2. Related Work

### 2.1. Crowd Counting and Localization

Crowd counting has evolved from traditional detection-based methods to modern density map regression approaches. Early works relied on hand-crafted features and sliding window detectors, which struggled with occlusions and scale variations. The seminal work of MCNN [1] introduced multi-column CNNs with different receptive fields, marking the transition to deep learning-based density estimation. Subsequent methods focused on improving density map quality through contextual information [5], attention mechanisms [6], and adversarial training [7]. Recent approaches have shifted toward point-based supervision and localization, with methods such as P2PNet [8] directly predicting point locations rather than density maps, offering more interpretable and accurate results. More recent task-specific transformer variants, including Gramformer [9] and CsViT [10], further strengthen long-range relation modeling or cross-scale interaction for counting and localization. Unlike these end-to-end transformer architectures, our goal is not to redesign the counting model from scratch but to adapt frozen foundation features so that they work inside the selective inheritance framework. Despite these advances, handling scale variation remains a persistent challenge that limits performance in real-world scenarios.

### 2.2. Scale-Aware Counting Methods

Addressing scale variation has been a central theme in crowd-counting research. Existing approaches can be broadly categorized into three paradigms: (1) Multi-scale feature fusion methods [2,11,12] aggregate features from multiple resolution levels or receptive fields. However, they typically apply uniform supervision across all scales, leading to mutual interference when the same object is counted at multiple resolutions. (2) Learn-to-scale methods [13,14,15] explicitly predict scale factors or density levels for image patches, then resize them for specialized processing. While effective, these approaches often require auxiliary supervision and multiple forward passes, increasing computational cost. (3) Selective inheritance methods, exemplified by STEERER [3], represent recent advances by introducing scale-customized feature selection and progressive inheritance. STEERER employs a Feature Selection and Inheritance Adaptor (FSIA) to disentangle scale-customized and scale-uncustomized features, combined with Masked Selection and Inheritance Loss (MSIL) that automatically determines the optimal scale for each region. Recent transformer-based or pre-trained-representation-based counting methods, such as CCTrans [16], CrowdFormer [17], and CrowdCLIP [18], further highlight the value of stronger global representations, but they do not study how frozen foundation features interact with selective inheritance across resolutions. In contrast to hybrid optimization-driven feature selection frameworks, our goal is not to design a new convergence mechanism but to calibrate frozen foundation features so that they can operate within the selective inheritance pipeline. The optimization protocol itself remains the inherited PWSP/MSIL training rule, whereas the new component is the DINO-specific feature calibration stage placed before that inherited protocol. Our work builds upon this paradigm but enhances it with foundation model representations.

### 2.3. Foundation Models for Dense Prediction

Vision foundation models pre-trained through self-supervised learning have demonstrated remarkable capabilities across various vision tasks. DINO [19] and its successor DINOv2 [20] learn visual representations through self-distillation with vision transformers, capturing rich semantic and structural information without manual annotations. DINOv3 [4] further improves upon this with enhanced training strategies and larger model capacity. These models have shown strong performance on dense prediction tasks, such as semantic segmentation [21,22] and depth estimation, often outperforming supervised pre-training. Recent works have explored adapting foundation models to specific tasks through parameter-efficient fine-tuning [23], prompt tuning [24], or lightweight decoder designs [25]. Hierarchical feature processing strategies [21,26] have proven effective for capturing multi-scale information in dense prediction scenarios. In the context of domain generalization, frozen foundation models have proven effective at preserving cross-domain priors [27,28], as end-to-end fine-tuning on limited source domains can lead to overfitting. Our work leverages DINOv3’s frozen representations for crowd counting, introducing task-specific adaptation through the Scale Harmonization Adapter while maintaining the model’s generalization capability.

## 3. Methodology

### 3.1. Overview

**Figure 1:**
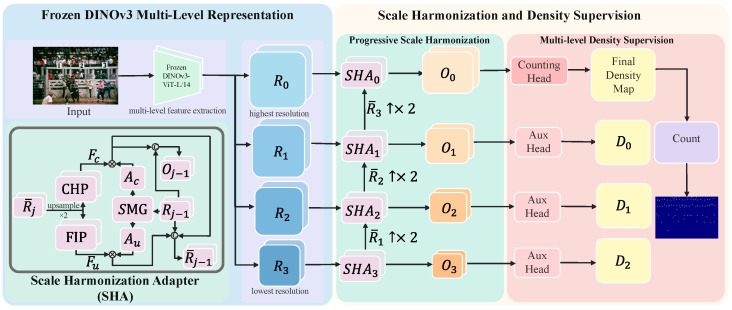
Overall architecture of D3-CalibCount. Intermediate features from selected DINOv3 blocks are aligned into a feature hierarchy and processed by cascaded Scale Harmonization Adapters (SHAs) for resolution-conditioned calibration. A shared counting head produces density predictions, while adapted features are progressively propagated across levels through selective inheritance.

Figure 1 illustrates the overall architecture of D3-CalibCount. Given an input image $I\in R^{H\times W\times 3}$, we first extract hierarchical intermediate features using a frozen DINOv3 backbone. Let ${\left\{R_{j}\right\}}_{j=0}^{N}$ denote the feature maps extracted from N+1 selected layers, where $R_{j}\in R^{C_{j}\times h_{j}\times w_{j}}$ represents the feature at the *j*-th resolution level with spatial dimensions $\left(h_{j},w_{j}\right)=\left(H/2^{j+2},W/2^{j+2}\right)$. Following the selective inheritance paradigm, we process these features from the lowest resolution $R_{N}$ to the highest resolution $R_{0}$ through a cascade of Scale Harmonization Adapters (SHAs). Inherited from STEERER are the coarse-to-fine processing order, the multi-resolution prediction hierarchy, and the training supervision built on PWSP/MSIL; newly introduced here are the frozen DINOv3 feature extractor, the SHA calibration modules, and the DINO-specific feature alignment that connects the backbone to this inherited framework. Each SHA module takes as input the current-level frozen features $R_{j-1}$ and the adapted features ${\bar{R}}_{j}$ from the previous (lower) resolution, producing scale-harmonized output $O_{j-1}$ for density prediction and inherited features ${\bar{R}}_{j-1}$ for the next level. The final prediction is generated from the highest resolution output $O_{0}$ through a lightweight counting head.

### 3.2. DINOv3 Feature Extraction

DINOv3 is a vision transformer trained through self-supervised learning with knowledge distillation. Unlike conventional supervised pre-training that optimizes for classification, DINOv3 learns to match the output distributions between a student and teacher network across different augmented views of the same image. This self-distillation process encourages the model to capture invariant visual representations that are robust to transformations while preserving fine-grained spatial details essential for dense prediction tasks.

We adopt the DINOv3-ViT-L/14 variant as our backbone, which consists of 24 transformer blocks operating on 14×14 pixel patches. For an input image I, the backbone first tokenizes it into a sequence of patch embeddings, then progressively refines these representations through self-attention layers. We extract intermediate features from blocks {5,11,17,23}, corresponding to different semantic levels: early layers capture low-level textures and edges, middle layers encode object parts and local structures, and deeper layers represent high-level semantic concepts. Importantly, we keep the entire DINOv3 backbone frozen during training, preserving its rich pre-trained representations and preventing overfitting to specific crowd-counting datasets.

The frozen backbone strategy offers several advantages: (1) Generalization: DINOv3’s diverse pre-training data ensures robust features that generalize across different crowd scenarios, camera viewpoints, and environmental conditions. (2) Efficiency: Freezing the backbone significantly reduces trainable parameters and memory consumption, enabling training on limited computational resources. (3) Stability: Frozen features provide a stable foundation for the adaptation modules, avoiding the instability often encountered when fine-tuning large models with small datasets. (4) Scale-awareness: DINOv3’s hierarchical architecture naturally encodes multi-scale information, with different layers being sensitive to different object sizes—a property we explicitly leverage through selective inheritance.

### 3.3. Scale Harmonization Adapter

The Scale Harmonization Adapter (SHA) serves as the bridge between DINOv3’s generic visual features and the specific requirements of scale-aware crowd counting. As illustrated in Figure 1, each SHA module operates at a specific resolution level *j* and consists of three learnable components: a Current-Resolution Harmonization Path (CHP), a Forward-Compatible Inheritance Path (FIP), and a Soft-Mask Generator (SMG). In intuitive terms, each SHA module decides which inherited evidence is already reliable enough to use at the current resolution and which evidence should be preserved and refined at the next finer resolution.

#### 3.3.1. Dual-Branch Architecture

The core design principle of SHA is to explicitly separate features into two streams based on their suitability for the current scale. Given the inherited features ${\bar{R}}_{j}$ from the lower resolution, we first upsample them to match the spatial dimensions of the current level: $R_{j}^{↑}=\mathrm{Upsample}\left({\bar{R}}_{j},h_{j-1},w_{j-1}\right)$. The upsampled features are then processed by two parallel branches.

**Current-Resolution Harmonization Path (CHP):** This branch, denoted as $C_{\theta_{c}}$, is designed to enhance features that are most discriminative for the scale range appropriate to the current resolution. It consists of two convolutional layers with batch normalization and ReLU activation:

(1) $$F_{c}=C_{\theta_{c}}\left(R_{j}^{↑}\right)={\mathrm{Conv}}_{3\times 3}\left(\mathrm{ReLU}\left(\mathrm{BN}\left({\mathrm{Conv}}_{3\times 3}\left(R_{j}^{↑}\right)\right)\right)\right)$$

The CHP learns to amplify features corresponding to objects whose scale matches the current resolution’s proficiency range, effectively acting as a scale-specific feature enhancer.

**Forward-Compatible Inheritance Path (FIP):** Complementary to CHP, the FIP $U_{\theta_{u}}$ processes features that may not be optimal for the current scale but could be valuable for higher resolutions. It shares the same architectural design as CHP but with independent parameters:

(2) $$F_{u}=U_{\theta_{u}}\left(R_{j}^{↑}\right)={\mathrm{Conv}}_{3\times 3}\left(\mathrm{ReLU}\left(\mathrm{BN}\left({\mathrm{Conv}}_{3\times 3}\left(R_{j}^{↑}\right)\right)\right)\right)$$

By maintaining this separate pathway, we prevent the premature discarding of potentially useful information, allowing the network to defer scale-specific decisions to higher resolutions where finer details become available. Intuitively, the dual-branch design separates two roles that a single branch would mix: CHP emphasizes evidence that is already reliable at the current resolution, whereas FIP preserves complementary evidence that may become more discriminative only after it is propagated to finer resolutions. This separation reduces the conflict between immediate prediction and future inheritance, which is the main conceptual reason we observe better scale alignment in practice.

#### 3.3.2. Soft-Mask Generation

The Soft-Mask Generator (SMG) $A_{\theta_{m}}$ dynamically determines the contribution of each branch based on local feature characteristics. It takes the upsampled features as input and produces a two-channel attention map:

(3) $$A=\mathrm{Softmax}\left(A_{\theta_{m}}\left(R_{j}^{↑}\right),\dim=0\right)\in R^{2\times h_{j-1}\times w_{j-1}}$$

where the softmax operation along the channel dimension ensures that the two attention channels $A_{c}$ and $A_{u}$ sum to one at each spatial location, creating a soft selection mechanism. The SMG consists of three convolutional layers that progressively refine the attention maps, learning to identify which regions contain objects at the appropriate scale for the current resolution. Using three stacked 3×3 convolutions gives the SMG a moderate local effective receptive field, which empirically produces smoother spatial masks than a shallower design while still preserving fine crowd boundaries; we therefore treat this as a practical design choice rather than a theoretically optimal setting. The soft mask serves as a local gate between CHP and FIP, so mixed-scale or ambiguous regions are not forced into a hard early decision and can retain complementary information for later resolutions. This continuous gating also avoids abrupt branch switching, which is the main intuitive reason it improves stability under mixed-scale regions. Since the present work is empirical rather than theoretical, we do not claim convergence guarantees or scale-selection optimality.

#### 3.3.3. Feature Fusion and Inheritance

The outputs of the dual branches are weighted by their corresponding attention maps and combined with the current-level frozen DINOv3 features $R_{j-1}$:

(4) $$O_{j-1}=\mathrm{Concat}\left(R_{j-1},A_{c}⊙F_{c}\right)$$

(5) $${\bar{R}}_{j-1}=\mathrm{Concat}\left(R_{j-1},A_{c}⊙F_{c}+A_{u}⊙F_{u}\right)$$

where ⊙ denotes element-wise multiplication, and Concat represents channel-wise concatenation. The output $O_{j-1}$ is used for density prediction at the current resolution, incorporating only the current-resolution harmonized features that are most relevant. Meanwhile, ${\bar{R}}_{j-1}$ serves as the inherited features for the next higher resolution, combining current-resolution harmonized and forward-compatible inheritance information to provide a comprehensive feature representation.

This design enables progressive feature refinement: at each resolution level, the SHA selectively extracts and forwards the most discriminative features for that scale while preserving complementary information for subsequent levels. The cascade of SHA modules creates a hierarchical feature inheritance pathway, where lower resolutions contribute their expertise on large objects to higher resolutions, which in turn focus on smaller objects while benefiting from the inherited large-object features.

### 3.4. Masked Selection and Inheritance Loss

To effectively train the Scale Harmonization Adapters, we adopt the Masked Selection and Inheritance Loss (MSIL) strategy from STEERER, which provides scale-specific supervision at each resolution level. The key insight is that each resolution should focus on objects within its proficient scale range, rather than attempting to count all objects accurately, regardless of size.

#### 3.4.1. Patch-Winner Selection Principle

We employ the Patch-Winner Selection Principle (PWSP) to automatically determine which resolution is most suitable for each image region. Intuitively, PWSP asks which resolution currently predicts each region best and uses that answer as a training-only supervisory cue. During training, each resolution level *j* produces a predicted density map $D_{j}^{\mathrm{pre}}$ through a shared counting head $E_{\theta_{e}}$:

(6) $$D_{j}^{\mathrm{pre}}=E_{\theta_{e}}\left(O_{j}\right)$$

The counting head is trained only on the final highest-resolution output and kept frozen for other resolutions, ensuring consistent density estimation patterns across scales. For each spatial location, we compute the per-pixel loss against the ground-truth density map $D_{j}^{\mathrm{gt}}$ across all resolutions, then select the resolution with minimum loss as the “winner” for that location:

(7) $$M_{j}^{g}\left(x,y\right)=\left\{\begin{array}{cc} 1, & \mathrm{if}\;j=\arg\min_{k}L\left(D_{k}^{\mathrm{pre}}\left(x,y\right),D_{k}^{\mathrm{gt}}\left(x,y\right)\right)\\ 0, & \mathrm{otherwise} \end{array}\right.$$

This creates a binary mask $M_{j}^{g}$ that identifies regions where resolution *j* performs best, effectively partitioning the image into scale-specific zones. Importantly, these winner masks are constructed only during training from prediction errors against the ground-truth density maps. During inference, no ground truth or additional scale-selection step is required. We therefore treat PWSP as a simple and interpretable supervisory heuristic inherited from the selective inheritance framework, rather than as a claim of optimal scale assignment. A limitation of PWSP is that it is a hard winner-take-all assignment, so regions near scale boundaries can receive noisy supervision; alternative soft weighting or attention-based scale-selection rules are possible but are beyond the scope of the present revision. For this reason, we use PWSP only as a training signal and rely on the continuous SHA features during inference.

#### 3.4.2. Selection and Inheritance Objectives

At each resolution *j*, we define two complementary loss terms. The selection loss $\ell_{j}^{S}$ encourages the current resolution to accurately predict objects within its proficient scale range:

(8) $$\ell_{j}^{S}=L\left(M_{j}^{g}⊙D_{j}^{\mathrm{pre}},M_{j}^{g}⊙D_{j}^{\mathrm{gt}}\right)$$

The inheritance loss $\ell_{j}^{I}$ ensures that the current resolution maintains the prediction quality achieved by lower resolutions on their proficient regions:

(9) $$\ell_{j}^{I}=L\left(\mathrm{Upsample}\left(M_{j+1}^{g}\right)⊙D_{j}^{\mathrm{pre}},\mathrm{Upsample}\left(M_{j+1}^{g}\right)⊙D_{j}^{\mathrm{gt}}\right)$$

The total loss at resolution *j* combines both terms:

(10) $$\ell_{j}=\ell_{j}^{S}+\lambda \ell_{j}^{I}$$

where λ is a balancing weight. The final training objective sums losses across all resolutions plus a final prediction loss on the highest resolution:

(11) $$L_{\mathrm{total}}=\sum_{j=0}^{N}\ell_{j}+L\left(D_{0}^{\mathrm{pre}},D_{0}^{\mathrm{gt}}\right)$$

This hierarchical supervision strategy provides several benefits: (1) Each SHA module receives explicit guidance on which features to select and enhance. (2) The inheritance loss creates a progressive constraint that ensures discriminative features from lower resolutions are preserved. (3) The automatic scale selection through PWSP eliminates the need for manual scale annotations or hyperparameter tuning.

### 3.5. Counting Head and Inference

The counting head $E_{\theta_{e}}$ is a lightweight module consisting of three convolutional layers that transform the scale-harmonized features into density maps. During training, it is optimized only on the highest-resolution output $O_{0}$ to learn the mapping from DINOv3-adapted features to crowd density. At inference time, we perform a single forward pass through the frozen DINOv3 backbone and the cascade of SHA modules, generating the final density map from $O_{0}$. The predicted count is obtained by integrating the density map: $\hat{N}=\sum_{x,y}D_{0}^{\mathrm{pre}}\left(x,y\right)$. For localization tasks, we apply non-maximum suppression on the density map to extract individual point locations.

## 4. Experiments

### 4.1. Experimental Setup

#### 4.1.1. Datasets

We conduct comprehensive experiments on three widely used crowd-counting and localization benchmarks to evaluate D3-CalibCount’s performance across diverse scenarios.

**ShanghaiTech** [1] consists of two parts: Part A contains 482 images with highly congested scenes (an average of 501 people per image), while Part B includes 716 images with relatively sparse crowds (an average of 123 people per image). This dataset is widely used for evaluating scale-variation handling due to its diverse density distributions and challenging perspective variations.

**UCF-QNRF** [29] provides 1535 high-resolution images with 1,251,642 annotations, featuring diverse perspectives and extreme density variations ranging from 49 to 12,865 people per image. The dataset presents significant challenges in handling very sparse and extremely dense crowds within the same evaluation protocol.

**JHU-Crowd++** [30] contains 4372 images with 1.51 million annotations, including challenging weather conditions and various viewpoints. It is split into training (2272 images), validation (500 images), and test (1600 images) sets. The dataset is particularly valuable for evaluating robustness to environmental variations and diverse imaging conditions.

These benchmarks collectively provide comprehensive coverage of different crowd-counting scenarios, from sparse to extremely dense crowds and from controlled environments to challenging real-world conditions, making them ideal for evaluating the effectiveness of our scale harmonization approach.

#### 4.1.2. Implementation Details

We implement D3-CalibCount using PyTorch 1.12.0 and train on NVIDIA A100 GPUs. The DINOv3-ViT-L/14 backbone is initialized with pre-trained weights from the official release and kept frozen throughout training. We extract features from transformer blocks {5,11,17}, and {23}, which cover early, intermediate, and late semantic stages of the backbone. Input images are resized such that the shorter side is 512 pixels while maintaining aspect ratio, with random horizontal flipping and color jittering for data augmentation.

The Scale Harmonization Adapters are randomly initialized and trained with the Adam optimizer using a learning rate of $1\times 10^{-4}$, weight decay of $1\times 10^{-4}$, and batch size of 8. We train for 2000 epochs on ShanghaiTech, 1500 epochs on UCF-QNRF, and 1000 epochs on JHU-Crowd++, with a learning rate decay by a factor of 0.5 every 200 epochs. The loss balancing weight λ is set to 0.5. For density map generation, we follow the standard practice of convolving point annotations with a Gaussian kernel whose size is adaptive to local crowd density.

The counting head consists of three 3×3 convolutional layers with channel dimensions {256,128,1}, using ReLU activation between layers. We use Mean Squared Error (MSE) as the base loss function L in all loss terms. Although the nominal number of epochs is large, only the SHA modules and the lightweight counting head are optimized, while the DINOv3 backbone remains frozen; this substantially reduces the overfitting risk compared with full-backbone fine-tuning and follows a stable training recipe inherited from the selective inheritance framework. At inference time, we perform single-scale testing without test-time augmentation.

#### 4.1.3. Evaluation Metrics

We adopt standard evaluation metrics for crowd counting: Mean Absolute Error (MAE) measures the average absolute difference between predicted and ground-truth counts, while Mean Squared Error (MSE) penalizes large errors more heavily. For localization, following common crowd localization practice, we use F1-score across multiple distance thresholds as the primary comparison metric and additionally report representative precision and recall at a standard threshold to aid interpretation. Additionally, we compute Normalized Absolute Error (NAE) to assess performance relative to crowd density.

### 4.2. Comparison with State-of-the-Art

#### 4.2.1. Results on ShanghaiTech

Table 1 presents the comparison with recent methods on the ShanghaiTech dataset. On Part A, which features highly congested scenes with scale variations, D3-CalibCount achieves an MAE of 57.0 and an MSE of 95.1. The improvements are also evident on Part B (MAE: 6.9, MSE: 10.6), where the relatively sparse crowds and larger object scales align well with DINOv3’s feature characteristics. Compared with the baseline STEERER-HRNet (Part A: MAE 60.1, MSE 97.1; Part B: MAE 7.7, MSE 10.9), our method shows consistent improvements, demonstrating the effectiveness of adapting frozen DINOv3 features for scale-aware counting. Compared with STEERER-HRNet, our method uses fewer trainable parameters in the adaptation part (28.4M vs. 65.8M), while its overall deployment cost is discussed separately in Table 2.

#### 4.2.2. Results on UCF-QNRF

UCF-QNRF’s high-resolution images and extreme density variations provide an ideal testbed for scale-aware methods. As shown in Table 3, D3-CalibCount achieves an MAE of 76.1 and an MSE of 116.8, showing improvements over the STEERER-HRNet baseline (MAE: 79.5, MSE: 134.1). The improvements are particularly evident in images with high density levels, where scale variation is the most severe. These results confirm that adapting frozen DINOv3 features can effectively handle challenging real-world scenarios.

#### 4.2.3. Results on JHU-Crowd++

On JHU-Crowd++, as presented in Table 4, our method achieves an MAE of 52.9 and an MSE of 235.6, demonstrating robust performance across diverse weather conditions and viewpoints. The consistent improvements across the benchmark datasets validate the effectiveness of adapting frozen DINOv3 features for scale-aware crowd counting.

#### 4.2.4. Localization Performance

For point-level localization, following common crowd localization practice, we evaluate on ShanghaiTech Part A primarily with F1-score under distance thresholds of $\sigma_{l}\in \left\{1,2,3\right\}$ pixels and report representative precision/recall at $\sigma_{l}=2$. D3-CalibCount achieves F1-scores of 0.768, 0.842, and 0.879 at the three thresholds, indicating improved density map quality. The high precision (0.815 at $\sigma_{l}=2$) indicates that our density maps accurately capture object locations, while the strong recall (0.871 at $\sigma_{l}=2$) demonstrates comprehensive detection of instances across all scales. We therefore view localization here as a supportive evaluation of density-map quality rather than the main contribution of the paper. Qualitative results show that D3-CalibCount produces cleaner density maps with sharper peaks at object locations, facilitating more accurate localization even in highly congested scenes with severe occlusions.

#### 4.2.5. Efficiency and Deployment Discussion

Table 2 reports deployment-oriented metrics under the same inference setting. Compared with STEERER-HRNet, D3-CalibCount retains a smaller trainable part but incurs a larger total model size and higher inference cost because the frozen DINOv3-L backbone dominates the forward pass. Under this setting, D3-CalibCount requires 1.484 TFLOPs, compared with 0.284 TFLOPs for STEERER-HRNet. Combined with the measured latency, FPS, and peak memory, this shows a clear accuracy-cost trade-off in deployment. We accordingly revise our claim from general “efficiency” to trainable-parameter efficiency and discuss the resulting deployment limitation more explicitly.

### 4.3. Ablation Studies

We conduct comprehensive ablation studies on UCF-QNRF and JHU-Crowd++ datasets to analyze the contribution of each component in our framework. Given the scope of the revision, we focus the ablation on backbone choice, adapter structure, and frozen-versus-fine-tuned training, which are the factors most directly tied to the paper’s main claim.

#### 4.3.1. Effect of DINOv3 Backbone

To isolate the contribution of DINOv3, we compare against STEERER using the same selective inheritance framework but with different backbones: VGG19, HRNet-W48, DINOv2, and DINOv3-ViT-L/14. As shown in Table 5, replacing HRNet with a frozen foundation backbone substantially improves performance. A direct comparison between DINOv2 and DINOv3 under the same SHA-based adaptation pipeline shows that DINOv3 performs better on UCF-QNRF and JHU-Crowd++, supporting our choice of backbone while keeping the rest of the framework fixed. At the same time, the gap between DINOv3 (w/o SHA) and DINOv3 (w/ SHA) provides empirical evidence that strong frozen foundation features alone are not fully aligned with selective scale-aware counting and that resolution-conditioned calibration remains necessary. Further incorporating SHA yields additional improvements, demonstrating the synergy between foundation model features and task-specific adaptation.

#### 4.3.2. Effect of Scale Harmonization Adapter

We conduct ablation experiments on the SHA design by comparing different configurations as shown in Table 6: (1) no adapter (direct use of frozen DINOv3 features), (2) single-branch adapter (only CHP), (3) dual-branch without soft-mask (equal weighting), and (4) full SHA. The progressive improvement validates each component’s contribution: the dual-branch architecture enables explicit separation of current-resolution harmonized and forward-compatible inheritance features, while the soft-mask generator provides adaptive feature selection based on local characteristics.

#### 4.3.3. Effect of Frozen vs. Fine-Tuned Backbone

To validate our design choice of freezing DINOv3, we compare against fine-tuning the entire backbone with a small learning rate ($1\times 10^{-5}$). Table 7 shows that freezing the backbone yields better test-set performance on UCF-QNRF and JHU-Crowd++. This suggests that parameter-efficient adaptation is sufficient to preserve the strengths of DINOv3 while avoiding the instability of full fine-tuning.

### 4.4. Qualitative Analysis

**Figure 2:**
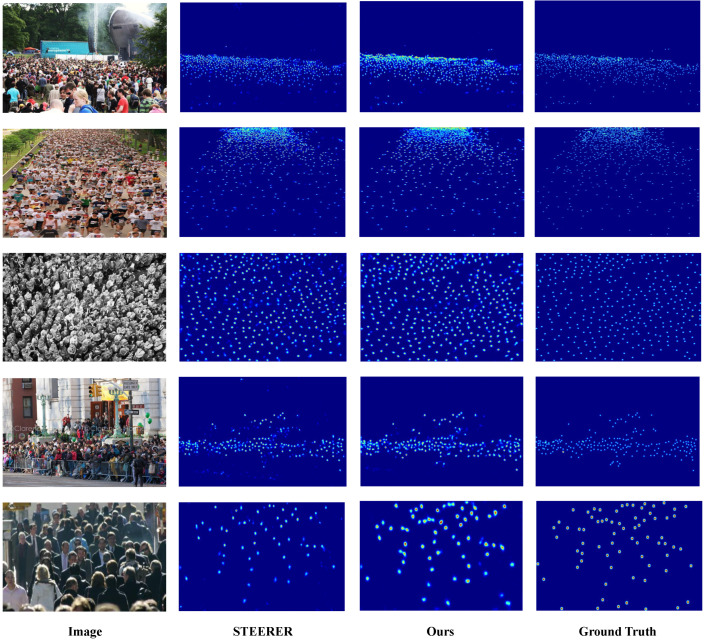
Qualitative comparison on ShanghaiTech Part A dataset.

**Figure 3:**
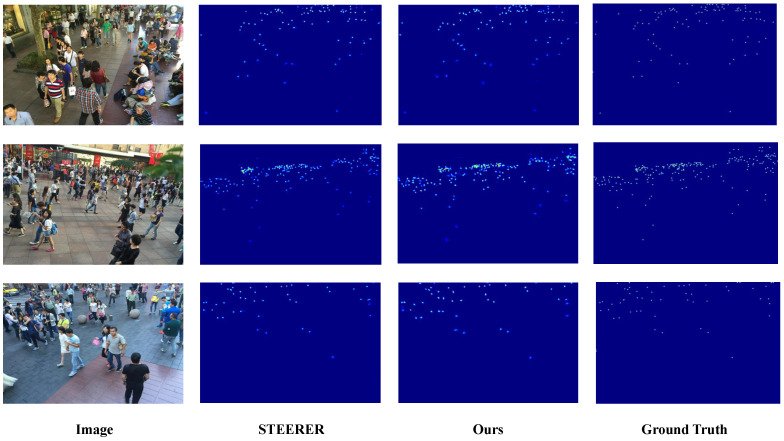
Qualitative comparison on ShanghaiTech Part B dataset.

**Figure 4:**
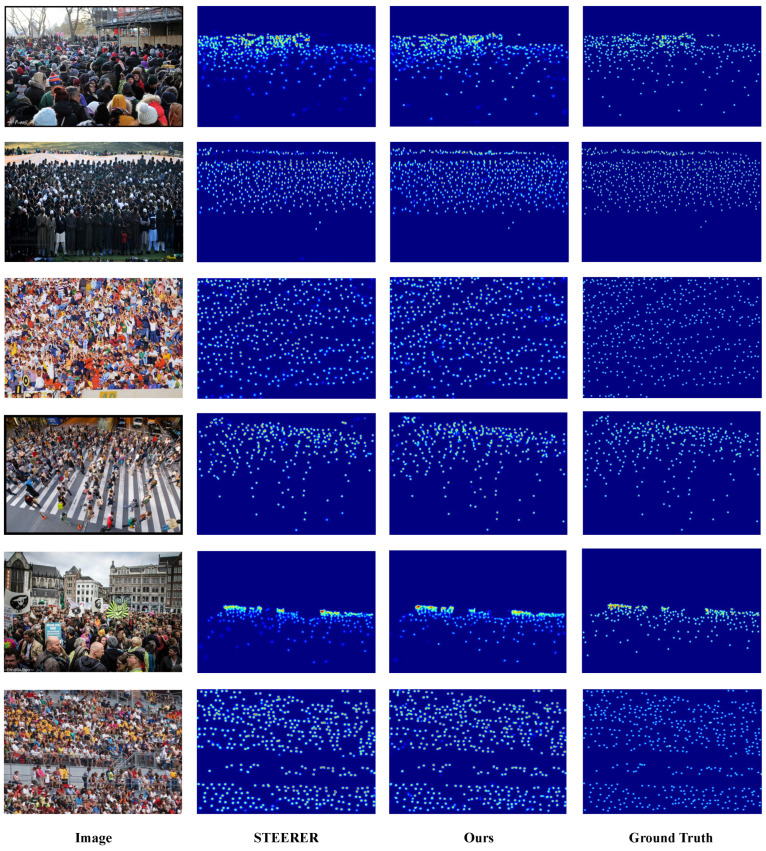
Qualitative comparison on UCF-QNRF dataset.

**Figure 5:**
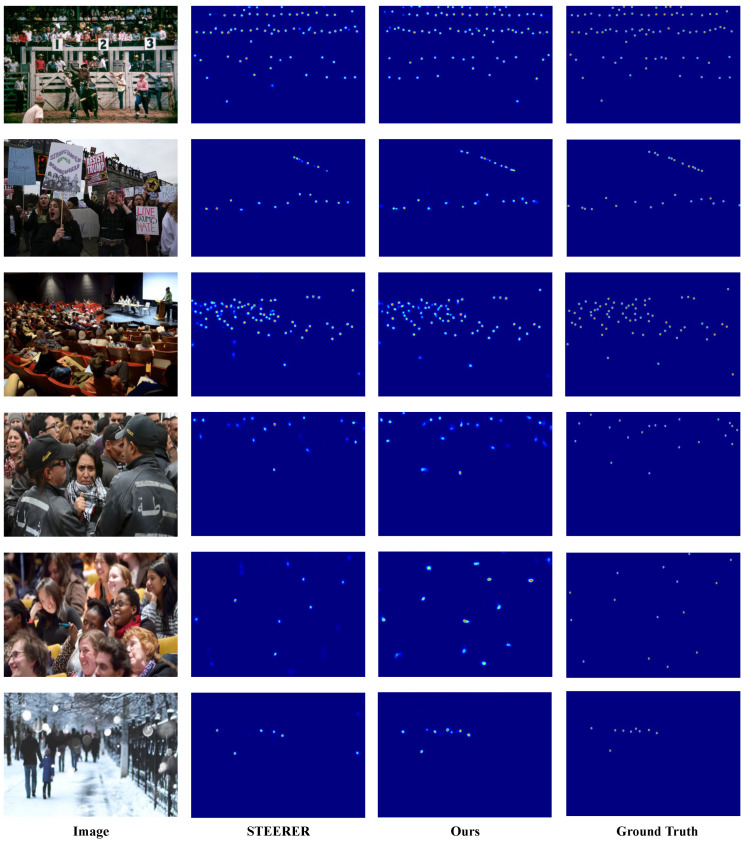
Qualitative comparison on JHU-Crowd++ dataset.

We present qualitative comparisons on the three benchmark datasets, with ShanghaiTech reported on Parts A and B, to demonstrate the effectiveness of D3-CalibCount in handling diverse crowd-counting scenarios. Figure 2, Figure 3, Figure 4 and Figure 5 show visual comparisons between our method and baseline approaches.

D3-CalibCount produces cleaner density maps compared with baseline methods, with more accurate localization of large and small objects. In scenes with severe perspective distortion, our method correctly identifies and counts distant small objects that are missed by other methods while simultaneously handling nearby large objects without over-counting. Qualitative inspection suggests that the soft-mask-guided scale-selection mechanism tends to emphasize coarse-scale regions at lower levels and dense small-instance regions at higher levels. This adaptive scale selection demonstrates the effectiveness of the proposed harmonization strategy.

### 4.5. Limitations and Deployment Scope

The present model is better suited to GPU-backed intelligent surveillance nodes than to ultra-light edge devices. Although freezing DINOv3 reduces the trainable portion of the network, the large backbone still increases total parameters, latency, and memory footprint during inference. In addition, severe perspective distortion, domain shift, or sensor noise can weaken the consistency assumption behind progressive inheritance, occasionally leading to underestimation in extremely dense distant regions or over-smoothing in sparse foreground regions. The present comparison scope is still limited to standard crowd-counting benchmarks and a focused set of ablations on backbone choice, adapter design, and freezing strategy; broader comparisons against additional foundation backbones, robustness protocols, or alternative scale-selection mechanisms remain future work.

## 5. Conclusions

In this paper, we investigated how to adapt frozen DINOv3 features for scale-aware crowd counting and localization. We showed that directly reusing generic foundation features is insufficient for selective scale-aware density estimation, and introduced the Scale Harmonization Adapter to perform resolution-conditioned feature calibration. Experiments on standard crowd-counting benchmarks demonstrate that the proposed design consistently improves over selective inheritance baselines while remaining parameter-efficient. The revised experiments also show a clear accuracy-cost trade-off: frozen foundation-feature adaptation improves counting accuracy and preserves training stability, but its current DINOv3-L instantiation is better suited to camera-based intelligent surveillance sensing at GPU-backed monitoring nodes than to strict edge-device deployment. These results suggest that lightweight adaptation of frozen foundation features is a practical direction for crowd counting and other dense prediction tasks requiring scale-sensitive reasoning.

## Figures and Tables

**Table 1:** Crowd counting performance on the SHHA and SHHB datasets.

Method	SHHA		SHHB	
	MAE	MSE	MAE	MSE
CAN [6]	62.3	100.0	7.8	12.2
SFCN [31]	64.8	107.5	7.6	13.0
S-DCNet [32]	58.3	95.0	6.7	10.7
BL [33]	62.8	101.8	7.7	12.7
ASNet [34]	57.8	90.1	-	-
AMRNet [35]	61.5	98.3	7.0	11.0
DM-Count [36]	59.7	95.7	7.4	11.8
GL [37]	61.3	95.4	7.3	11.7
D2CNet [38]	57.2	93.0	6.3	10.7
ChfI [39]	57.5	94.3	6.9	11.0
STEERER-HRNet [3]	60.1	97.1	7.7	10.9
D3-CalibCount (ours)	57.0	95.1	6.9	10.6
Improvement	↓ 3.0 (5.0%)	↓ 2.0 (2.1%)	↓ 0.8 (10.4%)	↓ 0.3 (2.9%)

**Table 2:** Efficiency comparison with STEERER-HRNet under the same inference setting.

Model	Total Params	TFLOPs	Avg. Latency	FPS	Peak Memory
STEERER-HRNet	64.64 M	0.284	32.130 ms	31.123	450.082 MB
D3-CalibCount (ours)	318.75 M	1.484	44.223 ms	22.613	1726.447 MB

**Table 3:** Crowd counting performance on the UCF-QNRF dataset.

Method	MAE	MSE
CAN [6]	107.0	183.0
SFCN [31]	102.0	171.4
S-DCNet	104.4	176.1
BL [33]	88.7	154.8
ASNet [34]	91.6	159.7
AMRNet [35]	86.6	152.2
DM-Count [36]	85.6	148.3
GL [37]	84.3	147.5
D2CNet [38]	81.7	137.9
P2PNet [8]	85.3	154.5
SDA+DM [40]	80.7	146.3
MAN [41]	77.3	131.5
ChfI [39]	80.3	137.6
RSI-ResNet50 [42]	81.6	153.7
STEERER-HRNet [3]	79.5	134.1
D3-CalibCount (ours)	76.1	116.8
Improvement	↓ 3.3 (4.2%)	↓ 17.3 (12.9%)

**Table 4:** Crowd counting performance on the JHU-CROWD++ dataset.

Method	MAE	MSE
CAN [6]	100.1	314.0
SFCN [31]	77.5	297.6
BL [33]	75.0	299.9
D2CNet [38]	73.7	292.5
SDA+DM [40]	59.3	248.9
MAN [41]	53.4	209.9
ChfI [39]	57.0	235.7
RSI-ResNet50 [42]	58.2	245.1
STEERER-HRNet [3]	57.4	247.4
D3-CalibCount (ours)	52.9	235.6
Improvement	↓ 4.6 (8.0%)	↓ 11.8 (4.8%)

**Table 5:** Ablation study on backbone networks. Results on UCF-QNRF and JHU-Crowd++.

Backbone	UCF-QNRF		JHU-Crowd++	
	MAE	MSE	MAE	MSE
HRNet-W48 (STEERER)	79.5	134.1	57.4	247.4
DINOv2 (w/ SHA)	77.2	122.5	54.5	239.5
DINOv3 (w/o SHA)	77.8	125.3	54.7	241.2
DINOv3 (w/ SHA)	76.1	116.8	52.9	235.6

**Table 6:** Ablation study on Scale Harmonization Adapter design. Results on UCF-QNRF and JHU-Crowd++.

Configuration	UCF-QNRF		JHU-Crowd++	
	MAE	MSE	MAE	MSE
No adapter	82.3	138.7	58.6	251.3
Single-branch (CHP only)	79.1	128.4	55.8	243.9
Dual-branch (w/o soft-mask)	77.4	121.6	54.2	238.7
Full SHA (ours)	76.1	116.8	52.9	235.6

**Table 7:** Ablation study on frozen vs. fine-tuned backbone. Results on UCF-QNRF and JHU-Crowd++.

Training Strategy	UCF-QNRF		JHU-Crowd++	
	MAE	MSE	MAE	MSE
Fine-tuned DINOv3	77.8	121.3	54.2	239.8
Frozen DINOv3 (ours)	76.1	116.8	52.9	235.6

## Data Availability

The data used in this study are publicly available from the corresponding benchmark datasets.
